# Attrition among New Zealand medical students completing research degrees: A 20-year analysis

**DOI:** 10.1007/s40037-019-00531-w

**Published:** 2019-08-22

**Authors:** Yassar Alamri, Tim J. Wilkinson

**Affiliations:** 1 New Zealand Brain Research Institute, Christchurch, New Zealand; 2 Department of Medicine, Christchurch Public Hospital, Christchurch, New Zealand; 3 Medical Education Unit, University of Otago, Christchurch, New Zealand

**Keywords:** Attrition, Physician-scientist, Career choice, Attitude of health personnel

## Abstract

**Introduction:**

Not all medical students who intercalate research degrees go on to completion. No study to date has investigated the specific reasons. Understanding this minority would fill an important research gap.

**Methods:**

A list was obtained of intercalating medical students who enrolled at our institution between 1995 and 2014. Students who withdrew from an intercalated research degree were then invited to complete an online survey via email.

**Results:**

Over the study period, 178 medical students commenced an intercalated honours or PhD degree with their medical degree, and 13 students withdrew from that program, giving an overall attrition rate of 7.3%. Students who withdrew from the intercalated degree were also more likely to withdraw from their medical degree (40%); this is compared with 3.6% of students who completed the intercalated degree, but eventually withdrew from their medical degree.

**Discussion:**

Demographics of this cohort were not dissimilar to those of completing students. Although withdrawing students had a higher exit rate from the medical degree, the rate of research involvement remained similar pre- and post-intercalation. The most commonly cited reasons for withdrawal were decreased satisfaction with research, and conflict with supervisors.

## What this paper adds

No study to date has investigated the specific reasons for the decisions of some medical students to complete or withdraw from intercalated research degree. Understanding this minority would fill an important research gap. The attrition rate of our intercalating medical students was relatively low (7.3%). Students who withdrew from the intercalated degree were also more likely to withdraw from their medical degree. However, there was no difference in attrition rates between male and female students, or those who published versus those who have not.

## Background

Concerns about the declining cohort of physician-scientists around the world have repeatedly been expressed in the literature [1]. The need for such qualified clinicians is paramount due to their unique position of seeing patients’ problems first-hand, while also collecting research data to inform better ways to solve those problems [2]. We have previously reviewed the available opportunities for medical students to undertake research, and found internationally that there are a multitude of opportunities afforded during their medical degree studies to complete research, either as core or elective [3].

Despite the need and the opportunities, not all students who embark on such scholarly activities go on to completion. Understanding the reasons for this is important if we are to enhance the attractiveness and effectiveness of such programs. The definition of attrition varies in the literature. For the purposes of the present study, attrition is defined as the withdrawal of a student from a research degree after initially commencing their study. Higher attrition rates are not only a concern for the student, but also for other stakeholders—governments and universities—due to the amount of resources invested in such ventures [1, 2, 4]. For a combined MD/PhD program specifically, international data indicate an attrition rate of around 10% [2].

The University of Otago is the oldest tertiary institution in New Zealand, and is considered a ‘research-intensive’ university with a very high submission rate (88%) for PhD students in the health sciences field (i.e., attrition rate of 12%) [5]. Medical students at the University of Otago are allowed to intercalate a research year which leads to the award of a Bachelor of Medical Science with Honours (BMedSc(Hons)) degree. If the student is interested and capable, they are given the opportunity to ‘upgrade’ to a Doctor of Philosophy (PhD), allowing them to graduate with a combined Bachelor of Medicine and Bachelor of Surgery (MBChB)/PhD degree [6]. Completion of the MBChB degree is a prerequisite for awarding the intercalated PhD [6]. On the other hand, students wishing to permanently exit from their MBChB degree after completing an intercalated BMedSc(Hons), may cross-credit prior study towards a health science-major Bachelor of Science (e.g., anatomy, physiology or pharmacology) [7]. These combined degree programs are the subject of the present research.

No study to date has investigated the specific reasons for attrition among medical students undertaking formalized research. Previous surveys of physician-scientists (i.e., after obtaining both medical and PhD degrees) have identified several factors associated with attrition from a research-oriented career. These included financial constraints, decreased satisfaction with research, change in career intentions and workplace bullying [1, 2, 4].

While most intercalating students go on to completion, this is not the case for all; understanding this minority who do not complete would fill an important research gap. Therefore, the aim of this study was to examine the rates and contributing factors to attrition in medical students undertaking either BMedSc(Hons) or MBChB/PhD degrees at our institution.

## Methods

### Study design and participants

A list of all medical students who enrolled for an intercalated degree between 1995 and 2014 was obtained from the Dean’s Office, Otago Medical School, Dunedin, New Zealand. A 4-year follow-up period was allowed to accommodate the current cohort of intercalating students. Students who withdrew from an intercalated research degree were then invited to complete an online survey via email. Two additional reminder emails requesting participation were sent in 6‑week intervals. This study was approved by the University of Otago Human Ethics Committee (reference D18/050) in accordance with the Declaration of Helsinki.

### Data collection

Participants were asked for details of their current position, scientific publications and career choices prior to as well as since intercalation. Based upon previous studies [2, 5], questions were specifically developed by the authors for the current study. The survey included questions on current medical/research careers (4 questions), and prior research experiences (3 questions). In addition, participants were asked to rank five barriers to undertaking an intercalated degree (from most to least applicable, with an additional ‘not applicable’ option). These barriers were extrapolated from information gained from the physician-scientist literature (financial reasons, decreased satisfaction with research, conflict with supervisors, change in career intentions, and bullying) [1, 2, 5]. Finally, participants were invited to respond to an open-ended question by writing in free-text about any other barriers not included in the list, as well as advice for prospective students (2 questions). Each participant was assigned a unique code in order to preserve anonymity and prevent data duplication.

### Data analysis

Descriptive statistics were used to present most of the data (expressed as means or proportions). Comparisons were made with two-sided Student’s t test (for continuous data), and Chi-square test (for categorical data). Any trends over time in attrition rates by year were compared using Spearman’s rank order correlation. All analyses were performed using SPSS Statistics® software package (version 22.0.0.0). Finally, free-text comments on the reasons for withdrawal and advice for future students were counted and summarized.

## Results

### Participants

Over the 20-year study period, 178 medical students commenced an intercalated BMedSc(Hons) or PhD with their medical degree, and 13 students withdrew from that program, giving an overall attrition rate of 7.3%. The mean attrition proportion (for any given year) did not significantly change over the study period (*r*_*s*_ = 0.3, *p* = 0.15). Due to the small sample size, and anonymity of responses, it was not possible to establish whether students intercalating a PhD had a higher or lower attrition rate compared with students intercalating a BMedSc(Hons).

Of the 13 students who withdrew from an intercalated program, seven completed the survey, giving a response rate of 53.8%. However, demographic and publication data were available for all 13 students. Table 1 shows a comparison of completing [8] and withdrawing students.

**Table 1:** Comparison of students completing intercalated research degrees with students withdrawing

	Completed	Withdrew	*P*-value
Number	165	13	
Sex (% male)	76.9%	61.4%	0.2^b^
Mean age (years)	19.7	20.0	0.5^c^
MBChB entry route (% post-graduate)	7.5%	9.1%	0.8^b^
Peer-reviewed publication^a^ (%)	41%	33%	0.6^b^
Withdrawal from MBChB (%)	3.6%	40%	0.05^b^

*MBChB* Bachelor of Medicine and Bachelor of Surgery

^a^Refers to percentage of intercalating students who published any work (in an indexed journal) at any stage of their career up to June 2018

^b^Chi-square test

^c^Student’s unpaired t test

### Pre-intercalation career

The majority of students were admitted to the medical course after high-school (10 out of 11 known responses; 90.9%). In addition, the majority of students (11/13; 84.6%) chose to intercalate prior to commencing their clinical years. Three students (out of 7 known responses; 42.9%) had previous research experience (most commonly in the form of a summer research project) prior to embarking on the intercalated degree.

### Post-intercalation career

Exit from the MBChB program was more likely to occur in those who withdrew from the intercalated research degree (40%) compared with students who completed the intercalated research degree (3.6%; *p* = 0.05). Having published did not discriminate those who completed the intercalated degree (41%) from those who did not (33%; *p* = 0.6).

The reasons for withdrawal included: decreased satisfaction with research (*n* = 5), conflict with supervisors (*n* = 4), change in career intention (*n* = 2) and health-related concerns (*n* = 2). Despite this, since withdrawing from the intercalated program, five students (5/13; 38.5%) subsequently participated in other research activities. A summary of the free-text comments can be found in Table 2.

**Table 2:** Quotes from the survey respondents

*Perceived barriers*	Project-related	‘I stopped enjoying the practical requirements in my specific research’ *Participant 1*
		‘Early dead-end on original project. The replacement project was nebulous and poorly defined’ *Participant 2*
	Supervisor-related	‘Supervisor was not equipped to supervise me at my level’ *Participant 3*
		‘More support. Often would go weeks without seeing supervisor or anyone else to answer questions’ *Participant 4*
	Student-related	‘My main reason for not completing the project was health’ *Participant 4*
		‘Both lack of preparation from myself as well as my supervisor’ *Participant 5*
*Influence on career*	‘[It affected] negatively only in the sense that I currently am not doing research in a lab … So it showed me what I didn’t like’ *Participant 1*	
	‘I think I am now less likely to pursue a research career based on my experiences. The research work in [specialty] reinforced my decision to train in the area’ *Participant 2*	
	‘I was influenced by my postgraduate years rather than by the program’ *Participant 6*	
*Advice*	‘Have a student mentor who has recently finished the program’ *Participant 1*	
	‘Start ethics [application] in the summer previous or earlier even’ *Participant 3*	
	‘Wait until you have finished medical school before deciding whether you want to commit a few years to [PhD] research’ *Participant 6*	

## Discussion

The present study revealed an attrition rate of 7.3% among medical students completing an intercalated research degree at our institution between 1995 and 2014. This rate is lower than that reported in the literature—although a direct comparison may not be fully justified as the quoted 10% attrition rate comes from a national survey of MD/PhD students in the US [2].

We did not find a difference in attrition between male and female students; this is consistent with findings from the US [2]. However, women, on average, made up a smaller proportion of intercalating students: 42.5% of all BMedSc(Hons) students, 40% of all MBChB/PhD students (Alamri et al., unpublished) and 37% of US MD/PhD students [2], compared with 55–60% of all medical students at our institution [6].

From the available data, the proportion of withdrawing students engaging in other research activities before versus after intercalation was similar (around 40%). How this proportion differs from that of students who had completed their intercalated degree or those who never intercalated is unknown, and is the subject of an ongoing study.

Most studies in the literature on physician-scientist attrition have focused on the early- and mid-career workforce (i.e., after completion of the medical/research degrees). For example, Koike and colleagues reported up to 30% of Japanese physician-scientists had left such a career path during the survey period [9]. Notwithstanding, useful strategies to further reduce attrition among intercalating medical students in New Zealand may be extrapolated from studies on physician-scientists. For example, the establishment of a centralized oversight (institutional or national) of intercalating students may provide a unified channel through which support (e.g., financial, career development, and re-integration into medical course) is accessed. This scheme has been suggested to facilitate the growth of the Japanese physician-scientist workforce [8]. Strategies to facilitate and encourage more women physicians into academia have already been described in Australasia [10] and elsewhere [2, 4].

We found the main predictors of non-completion of a research degree to be: reduced satisfaction with research, factors related to re-considering medicine as a career (as evident by withdrawing from the medical degree), problematic student-supervisor relationships, and challenging research projects. Other factors (e.g., illness) were also found to impede the student’s progress, although these were unpredictable. Our findings of specific (and reasonably consistent) reasons for withdrawal appear to be novel as there is paucity in the literature exploring reasons for attrition among medical students. It is noteworthy that financial concerns (either current or anticipated) were not cited by any of the students responding to our survey.

While some obstacles cannot be anticipated or easily addressed (e.g., illness), others may be more amenable to intervention in order to curtail the loss of young talents. At our institution, PhD students and their supervisors are highly encouraged to sign a memorandum of understanding at an early stage of the research project so that clear expectations may be established. This measure is not fault-proof, as the document is neither a current requirement for MBChB/PhD students, nor is it routinely completed for BMedSc(Hons) students. However, if completed and regularly reviewed/updated, the memorandum ought to provide an early means of identifying any difficulties with the research progress (which could impact of the student’s satisfaction/motivation), and troubleshooting any identified conflicts between the student and supervisor(s) by eliciting the help of the advisory committee. Additionally, tertiary institutions ought to invest in resources needed for students to support the correct choice of supervisor(s), and to offer mentorship training for new advisors/supervisors.

The present study is not without limitations. The low number of withdrawing students combined with a 53.8% response rate (typical of medical education surveys [11]) has led to only a few participants being included. This could have led to an underpowered analysis. Because results are from a single institution, this limits the external validity of the findings. Using questionnaires may limit the content of responses, and force interpretation of responses without the ability to clarify ambiguity. However, given the novelty of our findings, they ought to be viewed as an impetus for further research in this area.

## Conclusion

In conclusion, we found an attrition rate of 7.3% of medical students enrolled in intercalated BMedSc(Hons) and PhD degrees. Demographics of this cohort were not dissimilar to those of completing students. The most commonly cited reasons for withdrawal were decreased satisfaction with research and conflict with supervisors. Whilst some counter-measures (e.g., memorandum of understanding) are in place, future efforts ought to closely ascertain reasons for withdrawal and trial problem-focused solutions. Given the limitations of the present study, further research addressing the weaknesses identified is warranted.
